# Beyond Access and Awareness: The Role of Patient Experience and Physician Communication in Cervical Cancer Screening Participation in Hungary

**DOI:** 10.3390/cancers18182999

**Published:** 2026-09-16

**Authors:** Richárd Tóth, Pál Sebok, Cosette Spanics, Dóra Szerencsés, Balázs Vida, Márton Keszthelyi, Balázs Lintner, Icó Tóth

**Affiliations:** 1 Department of Obstetrics and Gynaecology, Semmelweis University, 1082 Budapest, Hungary; 2 Mallow Flower Foundation, Lágymányosi str. 7, 1111 Budapest, Hungary; 3 Innovation Management Doctoral School, Óbuda University, 1034 Budapest, Hungary

**Keywords:** cervical cancer, screening, participation, barriers, motivators, patient experience, cross-sectional study

## Abstract

Background/Objectives: To evaluate cervical cancer screening participation among women in Hungary and to identify motivators, barriers, experiences, and social factors associated with screening attendance. Methods: A cross-sectional survey with nationwide recruitment was conducted between November 2023 and September 2024 using non-probability voluntary sampling through a patient-foundation campaign, social media and other online communication channels, and community-based distribution of paper questionnaires. The survey assessed cervical cancer screening participation, time since last screening, screening frequency, healthcare setting, screening-related experiences, and perceived motivators and barriers. Sociodemographic and lifestyle characteristics, self-rated health, and communication about screening within the social environment were also collected. Data was analyzed using descriptive statistics and subgroup comparisons. Associations were evaluated using chi-square tests with effect sizes reported as Cramér’s V. Responses to open-ended questions were analyzed using qualitative content analysis. Results: A total of 5196 women were included in the analysis. Overall, 59.8% reported screening within the past year, while 17.3% attended more than two years earlier and 3.5% never went for screening. Screening participation was highest among women aged 25–54 years and lower among younger and older age groups. Communication about screening within the social environment was associated with more recent and more regular attendance. The proportion reporting screening within the past year was highest among women aged 25–54 years and lower among younger and older age groups. Women who reported discussing cervical cancer screening within their social environment were more likely to report recent and regular screening attendance. Screening experiences were generally favorable, although physical and emotional aspects were rated less positively. The most frequently reported motivation for attending screening was consciousness/health awareness, whereas major barriers included negative healthcare experiences, limited access to services, cost of private care, fear of diagnosis, and time constraints. Conclusions: Cervical cancer screening participation in this sample was high but varied across demographic, experiential, and social factors. Age and communication about screening were the factors most strongly associated with screening behavior, while structural barriers and negative healthcare experiences were common answers for reduced participation.

## Simple Summary

Cervical cancer screening can prevent many deaths, but some women still do not take part regularly because of practical, emotional, and healthcare-related barriers. This study aimed to understand what the facilitators and barriers among Hungarian women might be that influence screening attendance. We also wanted to learn how personal experiences with healthcare and discussions within family or social circles relate to participation. Our findings may help researchers and healthcare professionals designing better strategies to improve screening participation, creating more patient-friendly care, and reduce avoidable barriers. By combining survey results with women’s own explanations, this research can support future efforts to increase screening success and improve cervical cancer prevention.

## 1. Introduction

Cervical cancer remains an important global public health problem, accounting for approximately 600,000 new cases and more than 300,000 deaths annually, despite the availability of effective preventive measures, including human papillomavirus (HPV) vaccination and cervical cancer screening. Although Hungary has a lower incidence than many high-burden countries, cervical cancer remains an important cause of cancer-related morbidity and mortality, with an incidence of 24.7 per 100,000 women and a mortality rate of 9.52 per 100,000 women, highlighting persistent challenges in prevention and early detection [1,2,3,4]. While HPV vaccination offers the long-term promise, screening remains the cornerstone of prevention among adult women and a central component of the World Health Organization’s global strategy to eliminate cervical cancer as a public health problem. However, the efficacy of screening programs depends not only on availability but also on sustained participation. Despite the existence of organized screening services in many countries, participation remains suboptimal; although accurate data are not available in Hungary, cervical screening coverage is estimated at approximately 50–60%, still below the ideal 70% WHO target [5,6,7].

In Hungary, cervical cancer screening is delivered through a dual system consisting of an organized, population-based screening program complemented by opportunistic screening. Women aged 25–65 years receive an invitation to participate in cytology-based screening every three years, while many women do not wait for the invitation and rather go for annual screening during routine gynecological visits outside the organized program. Although this mixed approach increases the overall number of screening examinations performed and increases the total health expenditure, it has not achieved the population coverage necessary to maximize the public health benefits of screening. Moreover, substantial disparities in participation persist across socioeconomic and geographic groups, suggesting that barriers extend beyond healthcare [3,8].

Participation in cervical cancer screening is a complex health behavior influenced by multiple interacting structural, individual, and social factors. Established barriers include limited access to healthcare services, socioeconomic disparities, competing life priorities, and psychological factors such as fear, embarrassment, and low perceived susceptibility to cervical cancer [6,8,9]. More recently, increasing attention has focused on healthcare-related experiences as potentially modifiable determinants of screening uptake [10]. Likewise, social influences, including discussions about screening within families and peer networks, educational attainment, and health literacy, have emerged as important determinants of screening behavior [11].

Previous studies from Hungary have primarily examined knowledge, awareness, and healthcare access as influencing factors of screening participation, whereas considerably less attention has been paid to healthcare provider–related experiences, social influences, and patients’ lived experiences of screening [12,13,14]. Consequently, a comprehensive understanding of the interplay between structural, behavioral, experiential, and social determinants remains limited. To our knowledge, this is the largest study with nationwide recruitment conducted in Hungary to comprehensively investigate cervical cancer screening participation by integrating quantitative assessment of screening practices, healthcare-related experiences, perceived motivators and barriers, and social influences with qualitative analysis of women’s own descriptions of factors influencing screening attendance. Identifying which women are less likely to participate, and understanding the modifiable barriers underlying non-participation, is essential for designing targeted interventions to improve screening uptake and reduce preventable cervical cancer morbidity and mortality.

## 2. Materials and Methods

### 2.1. Study Design and Participants

A cross-sectional survey with nationwide recruitment was conducted between November 2023 and September 2024 to evaluate cervical cancer screening participation and associated factors among adult women in Hungary.

Participants were recruited through the “We are looking for your motivation” campaign organized by the Mallowflower Foundation using social media platforms, the Foundation’s online communication channels, and community-based distribution of paper questionnaires. Participation was voluntary, anonymous, and uncompensated.

Women aged ≥18 years residing in Hungary were eligible to participate. Responses collected through the online platform and paper-based questionnaires were merged into a single database for analysis. No formal a priori sample size calculation was performed because the study was designed as an exploratory, nationwide cross-sectional survey using voluntary recruitment. Instead, all eligible responses collected during the predefined recruitment period were included in the analysis after application of the prespecified exclusion criteria. Responses collected through the online platform and paper-based questionnaires were merged into a single database for analysis. Repeated online submissions were technically restricted, and paper questionnaires were distributed through controlled community-based channels to minimize the possibility of duplicate participation. As the survey was anonymous and no personal identifiers were collected, complete individual-level verification of duplicate participation was not possible. Questionnaires with more than four unanswered items were excluded from the final analysis.

### 2.2. Questionnaire Development and Study Variables

The survey instrument was a structured, anonymous questionnaire developed in Hungarian by the Mallowflower Foundation in collaboration with a multidisciplinary expert panel specifically for this study. Questionnaire development followed an iterative process comprising a literature review, item generation, expert assessment of content validity, pilot testing, cognitive pretesting, and evaluation of internal consistency.

The multidisciplinary expert panel comprised two gynecologic oncologists and one representative of the Mallowflower Foundation, a Hungarian advocacy group specializing in gynecological cancers and HPV prevention. Draft items were developed based on published literature, international cervical cancer screening recommendations, and clinical experience. Before administration to the study population, panel members independently assessed each item for relevance, clarity, comprehensiveness, and clinical applicability Their recommendations were incorporated through iterative consensus meetings, providing evidence tothe content validity of the instrument.

Before the main study, face validity and comprehensibility were assessed through pilot testing among 20 adult women from the target population. Participants completed the questionnaire and provided feedback on the clarity, relevance, acceptability, and interpretation of the questions and response options. Cognitive pretesting was additionally used to identify ambiguities, misunderstandings, and potentially misleading wording. Feedback from the pilot and cognitive testing was incorporated into the final questionnaire, resulting in minor modifications to item wording, sequencing, and response options. Pilot data were used exclusively for questionnaire refinement and were not included in the final study cohort or subsequent analyses.

The final questionnaire comprised both closed- and open-ended questions. The primary outcomes were cervical cancer screening participation, assessed by time since the last screening examination and habitual screening frequency. Additional domains included screening-related experiences, perceived motivators for participation, barriers to screening attendance, and communication about cervical cancer screening within participants’ social environments. Two open-ended questions specifically explored reasons for screening participation or non-participation. The non-participation question was presented only to participants who reported never having attended cervical cancer screening and asked about the reasons for not attending. The participation question was presented to all other participants and asked why they attended screening. Screening experiences were assessed using five-point Likert-scale items covering healthcare provider attitude, communication, physical discomfort, emotional experience, and overall examination duration. For these items, responses ranged from 1 (most negative experience) to 5 (most positive experience), with higher scores indicating more favorable screening experiences. Motivational factors for cervical cancer screening were assessed separately according to healthcare setting using three-point Likert-scale items, with 1 indicating the lowest and 3 the highest perceived importance. Sociodemographic and lifestyle characteristics included age, place of residence, height, weight, body mass index (BMI), smoking status, physical activity, and self-rated health.

Items belonging to the same conceptual domain were evaluated jointly as multi-item domains. No reverse scoring was applied. Missing responses were not assigned a score or imputed, and analyses involving individual items or multi-item domains were based on the available responses. Internal consistency of the multi-item domains was assessed using Cronbach’s alpha. The screening experience scale showed high internal consistency (Cronbach’s α = 0.893), while the motivator scales for public and private healthcare settings showed acceptable internal consistency (Cronbach’s α = 0.787 and 0.778, respectively). These assessments support the content and face validity of the questionnaire and the internal consistency of the evaluated multi-item domains, but do not establish comprehensive psychometric validation.

### 2.3. Data Collection

Data were collected using Google Forms (Google LLC, Mountain View, CA, USA) and standardized paper questionnaires. The paper-based format was offered to facilitate participation among women with limited internet access, lower digital literacy, or a preference for completing a printed questionnaire, thereby reducing potential barriers associated with exclusively online data collection. Both formats contained identical questionnaire items, response options, and instructions. Paper questionnaires were independently digitalized before integration into the study database. No personal identifiers were collected, warranting complete participant anonymity.

### 2.4. Statistical Analysis

Statistical analyses were performed using R version 4.5.3 (R Foundation for Statistical Computing, Vienna, Austria) with R Commander as the graphical user interface. Pearson’s chi-square tests of independence for contingency tables were performed using the stats package. Effect sizes for categorical associations were quantified using Cramér’s V calculated with the rcompanion package. Categorical variables are presented as frequencies and percentages, whereas continuous variables are summarized using means and standard deviations (SD). The number of missing responses for each variable is presented separately in the univariable descriptive tables. Associations between participant characteristics and cervical cancer screening behavior, including time since the last screening examination and habitual screening frequency, were assessed using Pearson’s chi-square test. Variables examined included age group, place of residence, body mass index category, smoking status, physical activity, self-rated health, and communication about cervical cancer screening within the participant’s social environment.

Effect sizes were quantified using Cramér’s V and interpreted according to Cohen’s recommendations (0.1 = small, 0.3 = moderate, and 0.5 = large effect). For statistically significant contingency tables, adjusted standardized residuals were examined to identify cell-specific contributions to the observed associations. Residuals with an absolute value ≥1.96 were considered statistically significant.

Responses to Likert-scale items evaluating screening experiences and motivational factors were analyzed descriptively using the psych package and are presented as means, standard deviations, and response distributions.

Free-text responses were analyzed using inductive qualitative content analysis. Two investigators independently reviewed all responses and developed an initial coding framework through iterative reading of the data. Codes representing similar concepts were grouped into broader themes through consensus discussion, with arising disagreements resolved by a third investigator. The frequency of each thematic category was calculated to complement the quantitative findings. Representative quotations were purposively selected to illustrate the major themes. All quotations were translated from Hungarian into English by a professional language institute to ensure linguistic accuracy while preserving their original meaning.

The open-ended responses were analyzed as an exploratory component intended to contextualize the quantitative findings. Because the participation and non-participation questions were administered to different respondent groups according to screening history, the resulting themes were interpreted within their respective groups. Thematic frequencies describe the responses provided and should not be interpreted as population prevalence estimates.

All statistical tests were two-sided, and a *p* value < 0.05 was considered statistically significant. No multivariable regression analyses were performed because the primary objective was descriptive characterization of screening participation and exploration of bivariate associations.

## 3. Results

**Figure 1:**
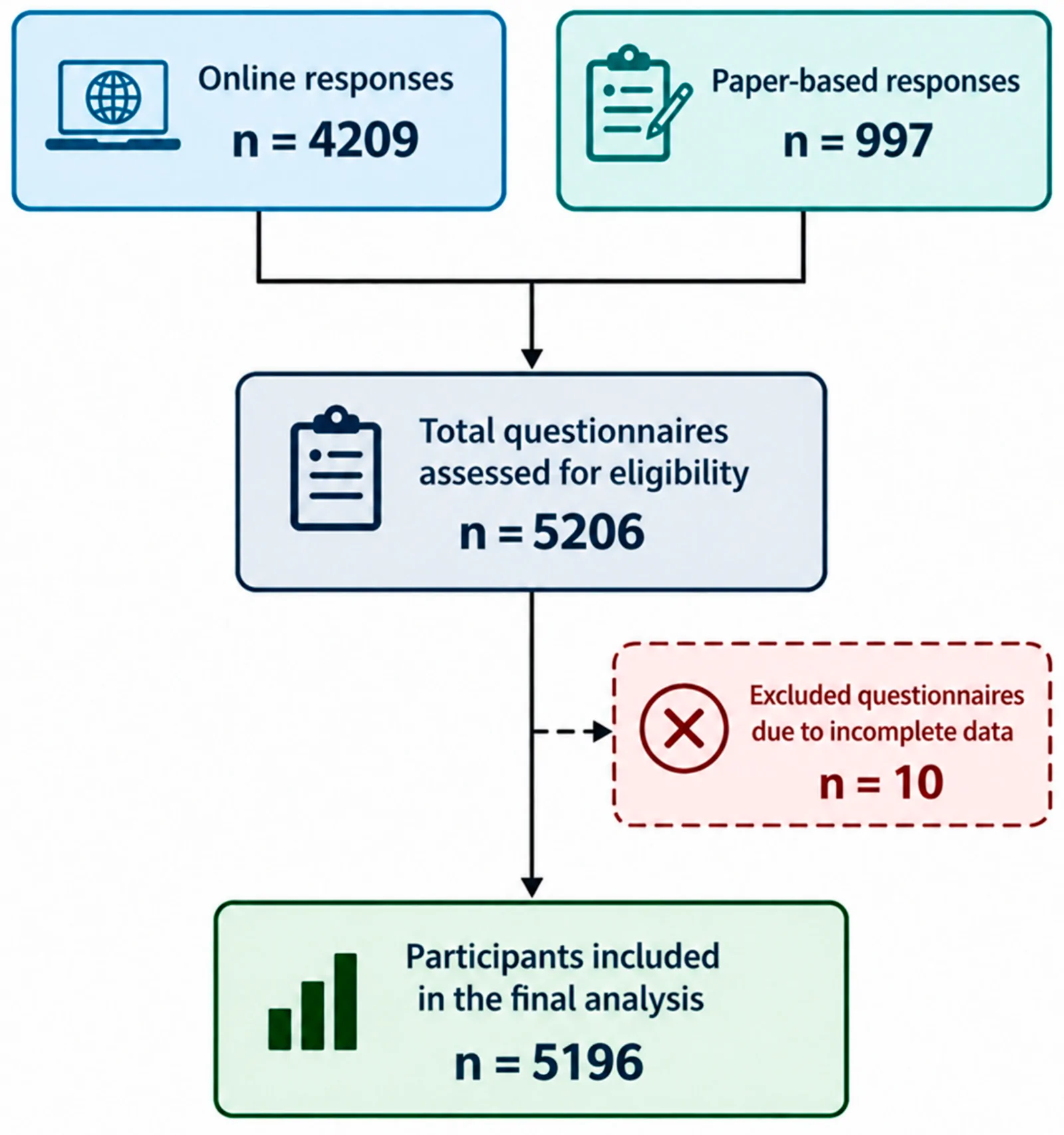
Flowchart describing the selection of the included questionnaires.

A total of 5206 questionnaires were received and assessed for eligibility. Ten questionnaires (0.2%) were excluded because of incomplete data, resulting in a final analytical sample of 5196 participants (Figure 1).

### 3.1. Baseline Characteristics of Participants

The mean age of the study population was 44.9 years, and the mean body mass index (BMI) was 26.3 kg/m^2^. More than two thirds of the participants rated their health as good or excellent while almost one-third perceived their health as moderate, and just a small minority of 3.2% reported poor health status (Table 1).

Participants were recruited from all over the country. 31.2% resided in smaller towns, a further 29.9% lived in regional cities and only one fifth originated from the capital. Villages were represented by one-sixth of the respondents.

**Figure 2:**
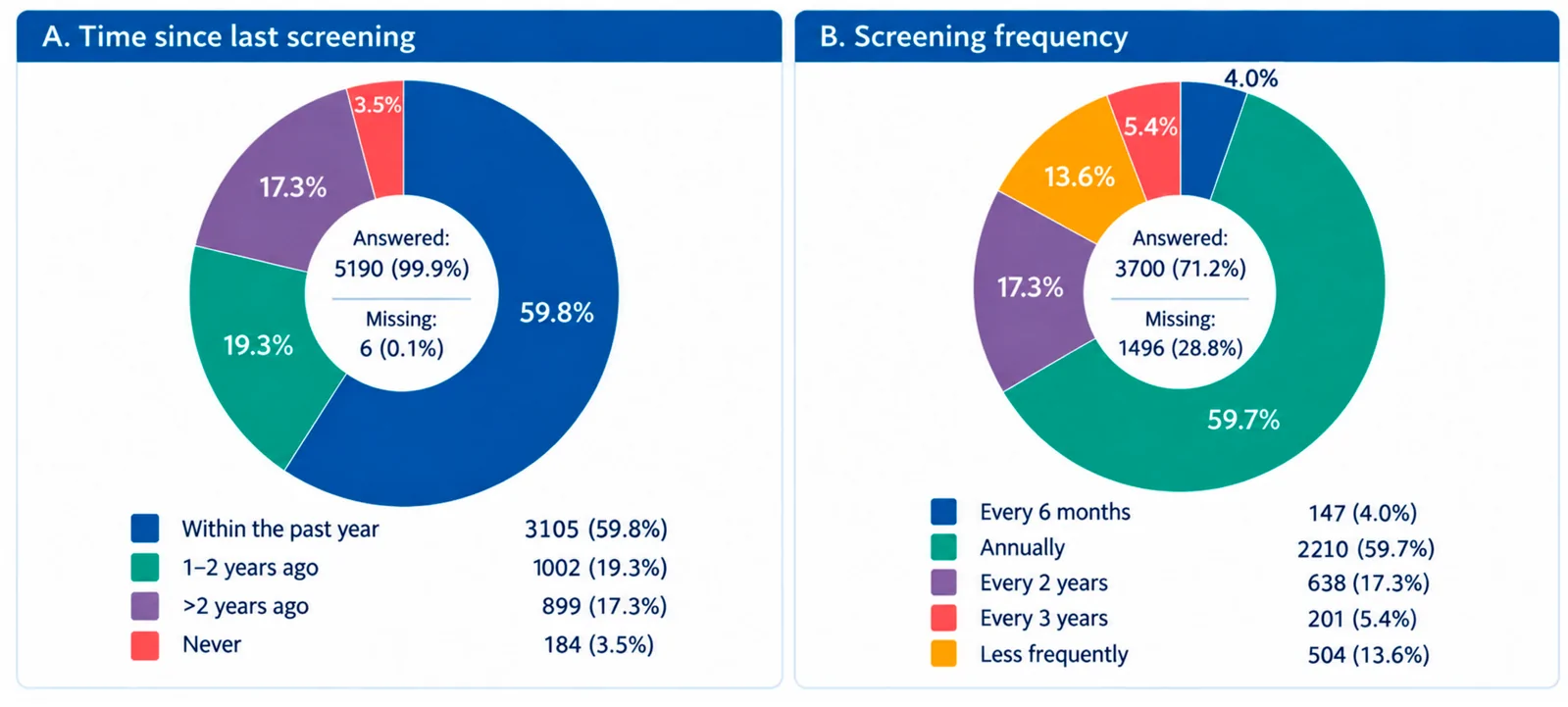
Screening behavior of the participants included in the final analysis. Note: Percentages are calculater based on available responses, with missin responses excluded from the denominator.

Regarding health-related behavior, vast majority of the participants were non-smokers. 31.5% of the participants were engaged in exercise more times per week, while 34.4% indicated no regular physical activity (Figure 2).

### 3.2. Cervical Cancer Screening Participation

Of all respondents, 98.9% correctly identified the method of cervical cancer screening. Screening-recency data were available for 5190 participants (99.9%), whereas screening-frequency data were available for 3700 of 5196 participants (71.2%) and missing for 1496 (28.8%). Regarding screening attendance, 59.8% (n = 3105) reported having undergone cervical cancer screening within the past year, while 19.4% had attended screening between 1 and 2 years. 17.3% reported that their last screening had occurred more than two years ago, and 3.5% (n = 184) had never participated in cervical cancer screening. 59.7% of the respondents attended screening once per year. Screening every 2 years and 3 years was reported by 17.3% and 5.4% of participants, respectively. An additional 13.6% reported less frequent participation.

Most participants, 74.3% (n = 3828) discussed screening within their social environment, whereas 25.7% indicated that the topic was not discussed (Table 2).

### 3.3. Experiences Influencing Cervical Cancer Screening

Participants generally reported positive experiences with cervical cancer screening (Table 3). The highest-rated aspect of the screening encounter was the attitude of the healthcare provider (mean 4.39, SD 0.94), followed by the duration of the examination (mean 4.34, SD 0.93) and the communication of the healthcare provider (mean 4.31, SD 1.01). Experiences related to the examination itself were less favorable. The emotional experience of the examination received a mean score of 3.84 (SD 1.17), with 36.8% of participants assigning the highest rating and 13.4% reporting negative experiences. The physical experience of the examination was rated lowest overall (mean 3.72, SD 1.10). Overall, participants expressed high levels of satisfaction with healthcare providers’ interpersonal skills and the duration of the screening visit, whereas the physical and emotional aspects of the examination were perceived less positively (Table 3).

### 3.4. Healthcare Setting and Motivational Factors for Cervical Cancer Screening

Overall, 52.1% (*n* = 2602) of participants reported attending cervical cancer screening in the private healthcare sector, while 47.9% (*n* = 2392) utilized public services (Table 4).

Among women attending public healthcare, the strongest motivations for participation were early detection of cervical cancer (mean 2.78, SD 0.47) and cancer prevention (mean 2.77, SD 0.48). Other commonly reported motivators included having the sample collected by a physician (mean 2.53, SD 0.67), the examination being free of charge (mean 2.52, SD 0.67), and the availability of screening within the local settlement (mean 2.48, SD 0.71), each of which was considered important by more than 60% of participants. In contrast, sample collection by a health visitor was the factor, least motivating (mean 1.58, SD 0.76).

Among women attending private healthcare, the most influential factor was short waiting time at the clinic (mean 2.34, SD 0.78), which was rated as important by 53.0% of respondents. By comparison, cost of the examination (mean 1.48, SD 0.56), ease of appointment booking (mean 1.48, SD 0.64), short waiting time for an appointment (mean 1.50, SD 0.67), and sample collection by a physician (mean 1.42, SD 0.68) received substantially lower importance ratings (Table 4).

### 3.5. Screening Frequency

All examined variables showed statistically significant associations with both time since last screening and screening frequency (*p* < 0.05 for all comparisons), with most Cramér’s V values below 0.10.

**Figure 3:**
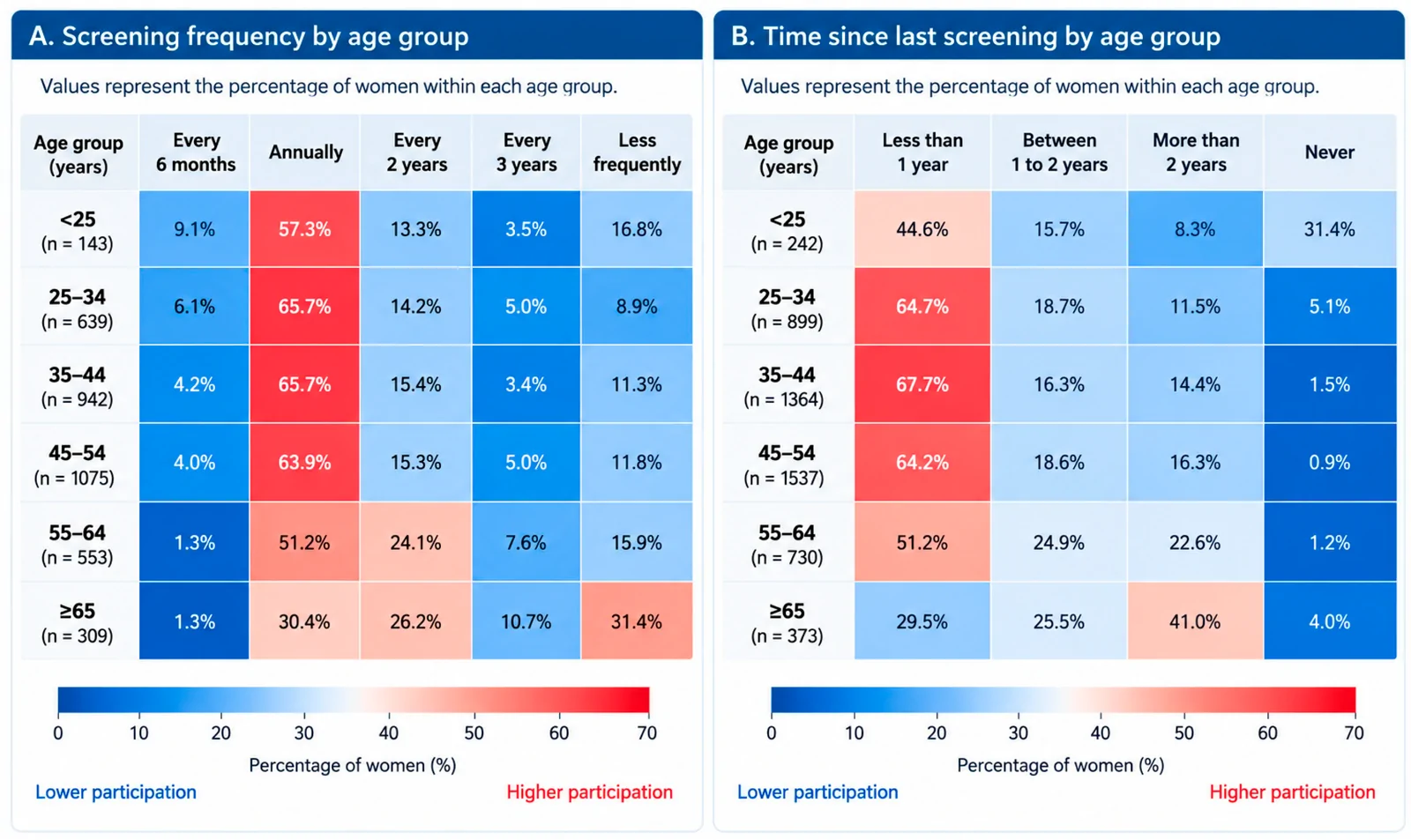
Heatmaps showing the association between age and screening behavior. (Percentages may not sum to 100% due to rounding).

The highest observed effect size was for age category in relation to time since last screening (χ^2^ = 894.16, *p* < 0.001, Cramér’s V = 0.241), representing a moderate effect size. Age category was also associated with screening frequency, although with a weaker effect (Cramér’s V = 0.132), with variation observed across age groups (Figure 3).

Communication about cervical cancer screening within the social environment was associated with both time since last screening (χ^2^ = 134.94, *p* < 0.001, Cramér’s V = 0.162) and screening frequency (χ^2^ = 87.62, *p* < 0.001, Cramér’s V = 0.155).

In contrast, other variables, including BMI, smoking status, physical activity, and place of residence, were associated with screening behavior only to a limited extent, with effect sizes ranging from 0.05 to 0.09, indicating limited magnitude of association (Figure 3) (Table S1).

### 3.6. Factors Associated with Time Since the Last Cervical Cancer Screening

The time elapsed since the last cervical cancer screening was significantly associated with self-rated health (χ^2^ = 82.35, *p* < 0.001, Cramér’s V = 0.073), age (χ^2^ = 894.16, *p* < 0.001, Cramér’s V = 0.241), discussion of cervical cancer screening within the social environment (χ^2^ = 134.94, *p* < 0.001, Cramér’s V = 0.162), place of residence (χ^2^ = 46.06, *p* < 0.001, Cramér’s V = 0.055), BMI (χ^2^ = 92.35, *p* < 0.001, Cramér’s V = 0.078), physical activity (χ^2^ = 82.01, *p* < 0.001, Cramér’s V = 0.073), and smoking status (χ^2^ = 27.31, *p* < 0.001, Cramér’s V = 0.073).

Among the examined factors, age showed the strongest association with screening recency. Screening within the previous year was most frequent among women aged 35–44 years (924/1364; 67.7%) and 25–34 years (582/899; 64.7%), compared with 29.5% (110/373) among women aged ≥65 years. Conversely, 41.0% (153/373) of women aged ≥65 years reported that their last screening had occurred more than two years previously, while 31.4% (76/242) of women aged <25 years had never undergone cervical cancer screening.

Screening recency also differed according to self-rated health. Screening within the previous year was reported by 66.3% (340/513) of women reporting excellent health and 62.6% (1807/2888) of those reporting good health, compared with 54.1% (835/1544) among those reporting moderate health. Among women reporting very poor health, 47.6% (10/21) had last been screened more than two years previously.

Differences were also evident according to social discussion of cervical cancer screening. Among women who discussed screening with family members, friends, or acquaintances, 63.9% (2446/3825) had been screened within the previous year, compared with 48.4% (640/1322) among those who did not. Conversely, screening more than two years previously was reported by 14.7% versus 24.7%, and never having been screened by 2.6% versus 6.2%, respectively.

Although statistically significant, the associations with place of residence, BMI, physical activity, and smoking status were small (Table S2).

### 3.7. Open-Ended Questions

A total of 2741 analyzable responses were obtained across the two open-ended questions addressing motivations for cervical cancer screening participation and reasons for non-participation or irregular attendance. Responses were analyzed using qualitative content analysis and categorized into seven thematic groups. The most frequently reported motivation was health consciousness, accounting for 1848 responses (67.4%), including maintaining health, disease prevention, and the importance of regular preventive examinations. Personal or family history of cervical cancer or other gynecological diseases was reported in 351 responses (12.8%), while fear of developing cervical cancer or delayed diagnosis accounted for 229 responses (8.4%). Other reported motivations included personal responsibility (74; 2.7%), physician recommendation or invitation (58; 2.1%), and family influence (51; 1.9%). Non-participation was reported in 130 responses (4.7%).

Overall, health consciousness was therefore the predominant motivation for screening participation, followed by personal or family history and fear. Among participants reporting non-participation or irregular attendance, the main barriers identified were negative healthcare experiences, limited accessibility of public services, the cost of private care, fear of a potential diagnosis, and time constraints. These qualitative findings are intended to complement the quantitative results by providing additional insight into participants’ self-reported motivations and barriers rather than serving as independent quantitative estimates (Table 5).

### 3.8. Qualitative Findings: Reasons for Non-Participation in Cervical Cancer Screening

Qualitative analysis was conducted using free-text responses to Question 9 (“What is the reason you have not attended or do not regularly attend cervical screening?”). The analysis included responses from participants who reported irregular or no participation in cervical cancer screening. Using inductive thematic content analysis, responses were coded and grouped into conceptually related categories. Representative quotations were purposively selected to illustrate the most prominent themes.

Among the 130 respondents identified as non-attenders, four principal themes emerged: negative experiences with healthcare providers, healthcare system-related barriers, fear, and low prioritization of screening.

The most frequently expressed barrier concerned negative experiences with healthcare professionals, particularly perceptions of disrespectful treatment, painful examinations, inadequate communication, and feelings of vulnerability during gynecological examinations. Several women described previous examinations as humiliating experiences that resulted in long-term avoidance of cervical screening, as follows:

*“I have had very humiliating experiences with medical examinations in general. I do not want to voluntarily place myself in a vulnerable situation. These experiences continue to resurface in everyday life, for example at work. I try to avoid trauma because I do not have emotional support, and I cannot let it affect my daily functioning. I can only rely on myself.”*

*“I felt like a piece of meat at the doctor’s office, as that is how I was treated during my last visit. Since then, I have not been able to bring myself to go again.”*

Another respondent described how a traumatic examination resulted in avoidance of gynecological care for almost two decades, but also illustrated how a later encounter with an empathetic physician restored her willingness to participate in screening:

*“I realized for the first time that gynecological examinations do not necessarily have to be traumatic and that the physician’s competence and attitude can make an enormous difference. That experience completely changed my perception of screening.”*

These narratives consistently highlighted the influence of physician communication, empathy, and respectful care on subsequent screening behavior.

A second major theme comprised healthcare system-related barriers. Participants commonly reported difficulties obtaining appointments within the public healthcare system and identified the financial burden of private healthcare as an obstacle to screening participation. Typical responses included:

*“Booking a suitable appointment is complicated; availability in public institutions is not always adequate, and private practices are expensive.”*

*“The public healthcare environment is unbearable, and private care is expensive.”*

These responses reflected both limited accessibility of publicly funded services and economic barriers associated with private care.

Fear also emerged as a recurring reason for non-participation. Respondents described anxiety about receiving an abnormal result or being diagnosed with cancer, which discouraged them from attending screening despite recognizing its importance. Representative comments included:

*“I do not go. It is extremely difficult to face the possibility of cancer.”*

*“It is irrational, but I am afraid of the result.”*

*“I have had many illnesses and surgeries, which have made things difficult, although this should not be an excuse.”*

Finally, many participants attributed their irregular attendance to low prioritization of screening, often related to competing family responsibilities, lack of time, forgetfulness, or seeking care only when symptoms occurred. Illustrative responses included:

*“Unfortunately, I cannot attend because of my small children.”*

*“Time flies, and I run with it. Family responsibilities take up all my time.”*

*“I only go if I have symptoms.”*

*“My own negligence.”*

Overall, the qualitative findings demonstrated that non-participation in cervical cancer screening was influenced by a combination of structural barriers, including healthcare accessibility and affordability, and individual-level factors, such as previous negative healthcare experiences, fear of diagnosis, and the low prioritization of preventive healthcare. The quotations further illustrate that the quality of interactions with healthcare professionals may have a lasting influence on future participation in cervical cancer screening.

## 4. Discussion

In this cross-sectional study, cervical cancer screening participation was generally high, with nearly 60% of women reporting screening within the past year. Screening behavior was associated with multiple factors, including self-rated health, age, and communication about screening within the social environment. However, while all examined variables showed statistically significant associations, effect sizes were generally small, most Cramér’s V values were below 0.10, indicating that most relationships were weak in magnitude.

The high frequency of self-reported annual screening is noteworthy, as the organized Hungarian program invites women aged 25–65 years to cytology-based screening every three years. However, annual gynecological follow-up is commonly recommended by gynecologists in routine practice, which may contribute to frequent opportunistic cytology outside the organized program. Other explanations include selection bias toward women engaged in preventive care, reporting of routine gynaecological visits rather than cytology specifically, and clinically appropriate closer surveillance in women with previous abnormalities or other risk factors. Thus, annual screening in this study cannot necessarily be considered inappropriate overscreening. Nevertheless, the findings suggest that frequent opportunistic testing may coexist with underuse of organized screening among other women, with implications for resource allocation.

Age demonstrated the strongest association with screening behavior, particularly regarding time since last screening, with higher participation observed among women aged 25–54 years and lower uptake among both younger and older age groups but these remained observational associations and should not be interpreted as large or causal effects. Communication about screening within the social environment also emerged as a consistently relevant factor, showing a weak but meaningful association with both screening frequency and recency.

Differences in reported motivational factors were observed between public and private healthcare users; however, these descriptive contrasts should not be interpreted as demonstrating independent service-preference drivers. The observed sector-specific differences may partly reflect socioeconomic and geographic stratification between public and private healthcare users. Qualitative findings supported these results, identifying health consciousness as the most common motivator and negative healthcare experiences, structural barriers, and fear as key contributors to non-participation.

The findings of the present study are consistent with existing evidence highlighting the importance of sustained screening participation for cervical cancer control. Recent epidemiological evidence further emphasizes the substantial global and regional disparities in cervical cancer incidence and mortality, as well as in access to and uptake of HPV-based screening and preventive interventions, underscoring the importance of equitable and sustained participation in screening programs [15]. High-performing systems demonstrate that sustained screening participation is critical for disease control and eventual elimination, as illustrated by the Australian experience, where high coverage and integration with vaccination programs have resulted in substantial reductions in incidence [16,17].

In contrast, multiple studies from low- and middle-income settings consistently report low screening uptake despite varying levels of awareness, highlighting persistent gaps between knowledge and participation. For example, screening coverage as low as 10–13% has been reported in community-based populations, often accompanied by substantial losses along the care cascade and systemic barriers to follow-up. These findings align with the present study in demonstrating that awareness alone is insufficient to ensure participation [3,18,19].

The association between self-rated health and screening participation observed in this study is consistent with evidence indicating that engagement in preventive behaviors is strongly linked to individual health perception and attitudes [19]. Similarly, the age-related disparities identified here correspond to patterns reported in other populations, where both younger and older women are less likely to participate in screening programs [8,20].

Importantly, the role of healthcare experience identified in this study supports existing evidence that patient–provider interaction is an important influencing factor of screening adherence. Negative experiences, including discomfort, fear, or perceived lack of empathy, have been consistently reported as barriers, while positive interactions facilitate continued participation [5,10,21]. In parallel, structural barriers such as cost, access, and waiting times remain important across different healthcare systems [5,11,22].

A notable finding of the present study is the association between communication about screening within the social environment and screening participation. While this factor has received comparatively less emphasis in the literature, related evidence suggests that targeted education and community-based interventions can substantially improve screening uptake. For example, a randomized controlled trial demonstrated that culturally tailored education and navigation significantly increased screening participation in underserved populations [23,24,25]. However, the cross-sectional design precludes determination of the direction of this relationship. Social communication may encourage screening, previous screening experiences may prompt subsequent conversations, or both may be related to unmeasured factors such as health consciousness, education, healthcare engagement, or access to care. Consequently, social communication should be interpreted as a correlate of screening behavior rather than an independently established determinant. Longitudinal and intervention studies are required to determine whether strengthening social or community communication results in increased participation [26,27].

The advances in screening technology further reinforce the importance of participation. HPV-based screening provides superior risk stratification compared with cytology, with substantially lower cancer risk following a negative test; however, residual risk remains, emphasizing the continued necessity of adherence to recommended screening intervals [28]. Furthermore on population-level participation has stronger effect on cervical cancer prevention than the type of screening method itself [29]. This underscores that even in settings with effective screening tools, participation remains a critical component of prevention.

Advances in screening are also extending beyond primary HPV detection to more refined risk-stratification strategies. P16/Ki-67 dual-stain cytology has emerged as a clinically useful triage approach for HPV-positive women and may improve the identification of individuals requiring further diagnostic assessment. Nevertheless, the population-level benefit of increasingly accurate screening and triage technologies continues to depend on equitable access and sustained participation in screening programs [30].

The qualitative findings complement the quantitative results by providing a more nuanced understanding of the barriers underlying non-participation in cervical cancer screening. While the survey demonstrated significant associations between screening attendance and several sociodemographic and lifestyle characteristics, the free-text responses revealed that women’s decisions regarding screening are also strongly influenced by previous healthcare experiences, emotional factors, healthcare system characteristics, and competing life priorities, which aligns with data available in the broader literature [9,22,31,32,33,34].

Perhaps the most striking finding was the prominence of negative healthcare experiences as a reason for avoiding cervical cancer screening. Many participants described previous gynecological examinations as painful, humiliating, or emotionally distressing, with some reporting that these experiences resulted in prolonged avoidance of screening. These narratives support the observation found in the literature that the consequences of negative clinical interactions may extend far beyond a single consultation and can substantially influence future preventive healthcare behavior [31,33,34,35,36,37,38,39].

However, the evidence concerning the persistence and magnitude of this relationship is not uniform. In a population-based survey from England, embarrassment and fear of pain were frequently endorsed, but neither independently predicted overdue screening after adjustment, whereas practical barriers such as appointment difficulties were more strongly associated with attendance [40]. Similarly, a study among migrant women in Portugal found that perceived pain, embarrassment, and previous negative screening experiences were commonly reported but were not independently associated with screening attendance; low perceived need and lack of motivation showed stronger associations with non-attendance [41]. Earlier survey data also indicated that, despite reported pain, embarrassment, or anxiety, 95% of women younger than 60 years intended to attend a subsequent cervical screening examination [42]. These findings indicate that an uncomfortable examination does not inevitably result in sustained screening avoidance and that its behavioral relevance may depend on perceived screening necessity, health literacy, access to care, and the wider social context. Accordingly, the narratives identified in our study should be interpreted as possible pathways contributing to avoidance rather than evidence of a durable causal effect.

These findings are consistent with growing evidence emphasizing the importance of trauma-informed and patient-centered care in gynecology [43]. Previous studies have shown that respectful communication, informed consent, preservation of patient autonomy, and empathetic examination techniques reduce anxiety, improve patient satisfaction, and increase willingness to participate in future gynecological care [44,45,46]. Conversely, insensitive communication, painful examinations, or perceived loss of control may contribute to long-term avoidance of preventive services [47]. The present findings suggest that improving interpersonal aspects of care may represent an important and modifiable strategy for increasing cervical cancer screening participation [48].

Structural barriers within the healthcare system also emerged as an important theme. Participants frequently reported difficulties obtaining appointments in the public healthcare system and identified the cost of private healthcare as a major obstacle. These findings align with previous studies demonstrating that accessibility of screening services—including appointment availability, waiting times, geographical accessibility, and financial barriers—substantially influences participation in organized and opportunistic screening programs [49,50,51]. Although Hungary provides publicly funded cervical cancer screening, regional inequalities in access and prolonged waiting times may encourage women to postpone or forego screening or seek care in the private sector when financially feasible.

Several respondents acknowledged avoiding screening because they feared receiving an abnormal cytology result or a diagnosis of cervical cancer. Similar findings have been reported internationally, where fear of cancer, anticipated pain, embarrassment, and anxiety regarding diagnostic procedures consistently emerge as psychological barriers to cervical cancer screening [51,52]. Importantly, fear appears paradoxical: although concern about cancer can motivate participation for some women, excessive fear may instead result in avoidance behavior, delaying diagnosis and treatment [53].

Low prioritization of preventive healthcare was also commonly reported. Women frequently attributed non-attendance to competing family responsibilities, lack of time, forgetfulness, or attending healthcare only when symptoms occurred. Aligning the international literature, these responses suggest that practical constraints and low perceived urgency remain important barriers despite widespread awareness of the benefits of screening [34,49,54]. Such findings support interventions including reminder systems, flexible appointment scheduling, workplace or extended-hour screening services, and educational campaigns emphasizing the asymptomatic nature of cervical precancerous lesions and the importance of regular screening irrespective of symptoms.

Overall, these findings suggest that improving cervical cancer screening participation requires a multifaceted approach. In addition to ensuring accessible and affordable screening services, interventions should promote respectful, patient-centered gynecological care, strengthen communication skills among healthcare professionals, reduce organizational barriers within the healthcare system, and address women’s emotional concerns regarding screening. Such strategies are likely to improve both the screening experience and long-term adherence to recommended cervical cancer screening intervals.

### 4.1. Strengths and Limitations

This study benefits from a large sample, nationwide recruitment, and complementary quantitative and exploratory free-text findings. However, its cross-sectional design precludes causal inference, and voluntary, non-probability recruitment may overrepresent health-conscious women, limiting national representativeness. Self-reported data are susceptible to recall and social-desirability bias. Questionnaire format was not retained in the analytical dataset, preventing comparisons between online and paper respondents and assessment of possible cross-mode duplication. Although questionnaire development included expert review, pilot and cognitive testing, and internal-consistency assessment, external validation was not undertaken. Analyses were predominantly bivariate, with potential residual confounding from incompletely measured socioeconomic and access-related factors; the large sample also warrants interpreting statistical significance alongside effect sizes. “Finally, the exploratory findings from the open-ended questions reflect only participants who provided free-text responses. These brief accounts offer limited interpretive depth and may not capture the full range of motivations or barriers. The different questions administered according to screening history also limit direct comparisons between respondent groups.”

### 4.2. Implications and Directions for Future Research

Future research should use probability-based sampling and linkage to screening registries to obtain more representative, objectively verified participation estimates. Longitudinal studies incorporating multivariable analyses should examine whether healthcare experiences and social communication predict subsequent screening attendance while accounting for socioeconomic circumstances, health literacy, and service accessibility. Prospective intervention studies should evaluate respectful, trauma-informed examination practices, clear provider communication, reminder systems, flexible appointments, and community-based support, using verified screening uptake as the principal outcome.

## 5. Conclusions

In this cross-sectional survey with nationwide recruitment, 59.8% of respondents with available screening-recency data reported screening within the previous year. Age showed the strongest bivariate association with screening recency, while social discussion and positive healthcare experiences showed smaller associations with screening behavior. Exploratory free-text responses provided context regarding motivations and barriers, including health consciousness, negative healthcare experiences, fear, and access difficulties. Overall, the findings support the evaluation of multifaceted interventions combining improved accessibility with respectful, patient-centered care and clear healthcare-provider communication. Community- and social-network-based approaches may also be promising, but their effectiveness should be evaluated prospectively. Future probability-based longitudinal studies using objectively verified screening records are needed to confirm these associations, distinguish appropriate surveillance from potential overscreening, and assess strategies to improve equitable participation.

## Figures and Tables

**Table 1:** Baseline characteristics of participants.

Variables	*N*	%
**Self-rated health status**		
Very poor	21	0.4%
Poor	145	2.8%
Moderate	1545	30.2%
Good	2889	56.6%
Excellent	513	10.0%
Missing answers	83	
**Place of residence**		
Capital city	1069	21.0%
County-level city	1521	29.9%
Town	1587	31.2%
Large village	160	3.1%
Village	730	14.3%
Unknown	9	0.2%
Abroad	16	0.3%
Missing answers	104	
**Smoking status**		
Yes	889	17.2%
No	4267	82.8%
Missing answers	40	
**Physical activity**		
Daily	338	6.6%
Several times per week	1284	24.9%
Weekly	904	17.5%
Monthly	855	16.6%
No physical activity	1774	34.4%
Missing answers	41	
	**Mean**	**SD**
Age (years)	44.9	12.6
BMI (kg/m^2^)	26.3	5.5

**Table 2:** Screening knowledge, behavior and screening-related experiences.

Category	*N*	%
**Method of cervical cancer screening**		
Blood test	15	0.3%
Cytology	5126	98.9%
Stethoscope	44	0.8%
Missing answers	11	
**Time since last screening**		
Within the past year	3105	59.8%
1–2 years ago	1002	19.4%
>2 years ago	899	17.3%
Never	184	3.5%
Missing answers	6	
**Screening frequency**		
Every 6 months	147	4.0%
Annually	2210	59.7%
Every 2 years	638	17.3%
Every 3 years	201	5.4%
Less frequently	504	13.6%
Missing answers	1496	
**Discussion of cervical cancer screening in social environment**		
Yes	3828	74.3%
No	1324	25.7%
Missing answers	44	

**Table 3:** Experiences of cervical cancer screening.

Variable	Mean (SD)	Distribution (n, (%)) *					Missing Answers (n)
		1	2	3	4	5	
Attitude of healthcare provider	4.39 (0.94)	86 (1.7%)	163 (3.3%)	583 (11.7%)	1022 (20.6%)	3119 (62.7%)	223
Communication of healthcare provider	4.31 (1.01)	111 (2.2%)	235 (4.7%)	622 (12.5%)	1022 (20.6%)	2978 (60%)	228
Physical experience of examination	3.72 (1.10)	233 (4.7%)	397 (8.0%)	1268 (25.6%)	1678 (33.8%)	1386 (27.9%)	234
Emotional experience of examination	3.84 (1.17)	283 (5.7%)	383 (7.7%)	1025 (20.6%)	1445 (29.1%)	1829 (36.9%)	231
Duration of examination	4.34 (0.93)	86 (1.7%)	132 (2.7%)	656 (13.2%)	1217 (24.5%)	2869 (57.9%)	236

* Likert scale values represent experiences from most negative (1) to most positive (5).

**Table 4:** Motivational factors for cervical cancer screening by healthcare setting.

Proportion of Respondents by Healthcare Setting					
	***N***	**%**			
Public healthcare	2392	47.9%			
Private healthcare	2602	52.1%			
Missing answers	202				
**Motivational factors (public healthcare users only)**					
**Variables**	**Mean (SD)**	**Distribution (n, %)**			**Missing** **answers (*n*)**
		**1**	**2**	**3**	
Free of charge in public healthcare	2.52 (0.67)	229 (9.6%)	690 (29.0%)	1458 (61.4%)	2819
Availability in local settlement	2.48 (0.71)	308 (13.0%)	614 (25.9%)	1450 (61.1%)	2824
Cancer prevention	2.77 (0.48)	67 (2.8%)	413 (17.4%)	1893 (79.8%)	2823
Early detection of cervical cancer	2.78 (0.47)	61 (2.6%)	394 (16.6%)	1925 (80.8%)	2816
Sample taken by physician	2.53 (0.67)	235 (9.9%)	647 (27.2%)	1493 (62.9%)	2821
Sample taken by health visitor	1.58 (0.76)	1351 (58.9%)	563 (24.6%)	379 (16.5%)	2903
**Motivational factors (private healthcare users only)**					
**Variables**	**Mean (SD)**	Distribution (n, %) *			**Missing** **answers (*n*)**
Cost of examination	1.48 (0.56)	1434 (55.3%)	1075 (41.5%)	82 (3.2%)	2605
Ease of appointment booking	1.48 (0.64)	1552 (59.9%)	829 (32.0%)	211 (8.1%)	2604
Short waiting time for appointment	1.50 (0.67)	1556 (60%)	781 (30.2%)	253 (9.8%)	2606
Sample taken by physician	1.42 (0.68)	1785 (68.9%)	523 (20.2%)	283 (10.9%)	2632
Short waiting time at clinic	2.34 (0.78)	497 (19.2%)	721 (27.8%)	1374 (53.0%)	2604

* Likert scale values represent motivators from least important (1) to the most important (3).

**Table 5:** Self-reported reasons for screening participation and non-participation.

Category	n	Percentage (%)
Non-participation	130	4.7
Personal/family history	351	12.8
Fear	229	8.4
Health consciousness	1848	67.4
Physician recommendation or invitation	58	2.1
Family influence	51	1.9
Responsibility	74	2.7
Missing answers	2455	

## Data Availability

Supporting data of this study are available from the corresponding author upon reasonable request.

## Supplementary Materials

The following supporting information can be downloaded at: https://www.mdpi.com/article/10.3390/cancers18182999/s1, Table S1: Distribution of cervical screening frequency according to participant characteristics; Table S2: Distribution of time since last cervical screening in relation to participant characteristics.

## Abbreviations

BMI	Body mass index
CI	Confidence interval
HPV	Human papilloma virus
SD	Standard deviation
WHO	World Health Organisation
