# Supplementary material for: Beyond Access and Awareness: The Role of Patient Experience and Physician Communication in Cervical Cancer Screening Participation in Hungary
Source: Cancers (Basel). 2026 Sep 16;18(18):2999. doi: 10.3390/cancers18182999 (PMC13604731; doi:10.3390/cancers18182999)
Supplement: Supplementary file 1 [file cancers-18-02999-s001.zip › cancers-4503371-supplementary.pdf]

Supplementary

Beyond Access and Awareness: The Role of Patient Experience and Physician Communication in Cervical Cancer Screening Participation in Hungary

Richárd Tóth[1$*], Pál Sebok[1$], Cosette Spanics[1,2], Dóra Szerencsés[3], Balázs Vida[1], Márton Keszthelyi[1], Balázs Lintner[1#], Icó Tóth[2#]

Informed Consent and Questionnaire

We Are Looking for Women's Motivation for Cervical Cancer Screening!

The Mallowflower Foundation is investigating women's motivation regarding cervical cancer screening.

What motivates or discourages you from participating in screening?

Participation in cervical cancer screening is low in Hungary, and our goal is to help improve this. We can only achieve this together, so we need your help.

Participation in this survey is **entirely voluntary**. You may choose not to participate or discontinue the questionnaire at any time before submitting your responses, without any consequences.

The questionnaire is **anonymous**. No personally identifiable information will be collected, and your responses cannot be linked to your identity. The collected data will be used solely for scientific research and may be published in aggregated form in scientific journals or presented at academic conferences. Individual responses will never be identifiable.

The questionnaire takes approximately **5–10 minutes** to complete.

By proceeding with this questionnaire, you confirm that:

you are **18 years of age or older**;

you have read and understood the information provided above;

you voluntarily agree to participate in this research study;

you understand that your anonymized responses will be used for scientific research purposes.

I have read the information above and voluntarily agree to participate in this study.

1. How is cervical cancer screening performed? Please select the correct answer.

A) Blood test

B) Cervical smear (Pap smear)

C) Endoscopy

2. When was your most recent cervical cancer screening?

A) Within the past year

B) 1–2 years ago

C) More than 2 years ago

D) I have never had cervical cancer screening

Please answer the following question only if you selected A, B, or C in Question 2.

3. How often do you attend cervical cancer screening?

□ Every 6 months

□ Every year

□ Every 2 years

□ Every 3 years

□ Less frequently

Please answer the following question only if you selected A, B, or C in Question 2.

4. What is the reason you attend cervical cancer screening?

(Please answer in your own words.)

Please answer the following question only if you selected A, B, or C in Question 2.

5. Please rate your experience during cervical cancer screening on a scale from 1 to 5.

(1 = very negative experience; 5 = very positive experience)

The attitude of the gynecologist/health visitor during the examination

① ② ③ ④ ⑤

Communication of the gynecologist/health visitor regarding the examination

① ② ③ ④ ⑤

Physical experience of the examination (pain, discomfort)

① ② ③ ④ ⑤

Emotional experience of the examination (embarrassment, fear, feeling of safety, trust)

① ② ③ ④ ⑤

Duration of the examination

① ② ③ ④ ⑤

Please answer the following question only if you selected A, B, or C in Question 2.

6. During your most recent cervical cancer screening, where did you receive care?

□ Public (free) healthcare

□ Private (paid) healthcare

Please answer the following question only if your most recent screening was in public (free) healthcare.

7. Please consider how much the following factors encourage you to attend cervical cancer screening.

(1 = does not encourage me at all; 2 = somewhat encourages me; 3 = strongly encourages me)

Screening is available free of charge within the public healthcare system

① ② ③

The service is available in your place of residence

① ② ③

Cancer prevention

① ② ③

Early detection of cervical cancer

① ② ③

The sample is collected by a physician in a specialist outpatient clinic

① ② ③

The sample is collected by a health visitor (public health nurse)

① ② ③

Please answer the following question only if your most recent screening was in private (paid) healthcare.

8. Please consider how much the following factors influence your decision to participate in cervical cancer screening.

(1 = does not influence me at all; 2 = somewhat influences me; 3 = strongly influences me)

Cost of the cervical cancer screening examination

① ② ③

Ease of scheduling an appointment

① ② ③

Ability to obtain an appointment within a short waiting time

① ② ③

The sample is collected by a physician in a specialist outpatient clinic

① ② ③

Short waiting time at the clinic

① ② ③

Please answer the following question only if you have never attended or do not regularly attend cervical cancer screening.

9. What is the reason you have never attended or do not regularly attend cervical cancer screening?

(Please answer in your own words.)

Please answer the following question only if you have never attended or do not regularly attend cervical cancer screening.

10. Under what circumstances would you consider attending or regularly participating in cervical cancer screening?

(Please answer in your own words.)

11. How would you rate your overall health?

□ Excellent

□ Good

□ Fair

□ Poor

□ Very poor

12. DEMOGRAPHIC INFORMATION

Remember: the questionnaire is anonymous!

Age: ____________________

Place of residence: ____________________

Body weight: ____________________

Height: ____________________

Do you smoke?

□ Yes

□ No

How often do you exercise?

□ Daily

□ Several times per week

□ Once a week

□ Once or twice per month

□ I do not exercise

13. Has cervical cancer screening ever come up in conversations with your friends or family?

□ Yes

□ No

Thank you for completing the questionnaire!

Supplementary Table S1. Distribution of cervical screening frequency according to participant characteristics

Category	Every 6 months	Annually	Every 2 years	Every 3 years	Less frequently
**Self-rated health**					
Very Poor	0 (0.0%)	5 (29.4%)	0 (0.0%)	3 (17.6%)	9 (52.9%)
Poor	6 (6.2%)	50 (51.5%)	13 (13.4%)	6 (6.2%)	22 (22.7%)
Moderate	51 (4.5%)	571 (50.5%)	226 (20.0%)	77 (6.8%)	206 (18.2%)
Good	79 (3.9%)	1292 (63.8%)	339 (16.7%)	100 (4.9%)	214 (10.6%)
Excellent	10 (2.8%)	251 (70.7%)	48 (13.5%)	10 (2.8%)	36 (10.1%)
**Age category**					
<25	13 (9.1%)	82 (57.3%)	19 (13.3%)	5 (3.5%)	24 (16.8%)
25-34	39 (6.1%)	420 (65.7%)	91 (14.2%)	32 (5.0%)	57 (8.9%)
35-44	40 (4.2%)	619 (65.7%)	145 (15.4%)	32 (3.4%)	106 (11.3%)
45-54	43 (4.0%)	687 (63.9%)	164 (15.3%)	54 (5.0%)	127 (11.8%)
55-64	7 (1.3%)	283 (51.2%)	133 (24.1%)	42 (7.6%)	88 (15.9%)
65+	4 (1.3%)	94 (30.4%)	81 (26.2%)	33 (10.7%)	97 (31.4%)
**Communication about cervical screening in social environment**					
Yes	116 (4.3%)	1700 (63.1%)	445 (16.5%)	145 (5.4%)	289 (10.7%)
No	29 (3.0%)	488 (50.5%)	188 (19.4%)	53 (5.5%)	209 (21.6%)
**Settlement type**					
Unknown	0 (0.0%)	3 (100.0%)	0 (0.0%)	0 (0.0%)	0 (0.0%)
Capital city	44 (5.4%)	519 (63.7%)	132 (16.2%)	35 (4.3%)	85 (10.4%)
Country-level city	35 (3.6%)	574 (58.8%)	170 (17.4%)	50 (5.1%)	148 (15.1%)
Town	38 (3.3%)	699 (60.6%)	205 (17.8%)	63 (5.5%)	148 (12.8%)
Large Village	6 (4.8%)	77 (62.1%)	20 (16.1%)	6 (4.8%)	15 (12.1%)

Village	20 (3.8%)	290 (54.7%)	89 (16.8%)	40 (7.5%)	91 (17.2%)
Abroad	2 (25.0%)	4 (50.0%)	2 (25.0%)	0 (0.0%)	0 (0.0%)
**BMI category**					
Underweight	3 (2.9%)	67 (64.4%)	16 (15.4%)	4 (3.8%)	14 (13.5%)
Normal	80 (5.0%)	1019 (63.1%)	266 (16.5%)	70 (4.3%)	179 (11.1%)
Overweight	38 (3.5%)	611 (56.4%)	208 (19.2%)	76 (7.0%)	151 (13.9%)
Obese I	15 (2.5%)	343 (58.0%)	111 (18.8%)	31 (5.2%)	91 (15.4%)
Obese II	7 (3.8%)	100 (54.9%)	26 (14.3%)	12 (6.6%)	37 (20.3%)
Obese III	3 (4.6%)	33 (50.8%)	5 (7.7%)	5 (7.7%)	19 (29.2%)
**Physical activity**					
Not regularly	34 (2.7%)	721 (56.9%)	229 (18.1%)	73 (5.8%)	210 (16.6%)
1–2 times a month	24 (3.9%)	347 (56.3%)	112 (18.2%)	35 (5.7%)	98 (15.9%)
Once a week	30 (4.8%)	421 (66.7%)	94 (14.9%)	38 (6.0%)	48 (7.6%)
Several times a week	47 (5.2%)	574 (63.7%)	156 (17.3%)	41 (4.6%)	83 (9.2%)
Daily	11 (4.4%)	127 (51.0%)	40 (16.1%)	9 (3.6%)	62 (24.9%)
**Smoking status**					
Non-smoker	120 (4.0%)	1832 (60.9%)	516 (17.2%)	159 (5.3%)	379 (12.6%)
Smoker	26 (3.9%)	354 (53.7%)	118 (17.9%)	39 (5.9%)	122 (18.5%)

Data is shown as absolute numbers and percentages in brackets, as n(%). Obese I if 30< BMI <35, Obese II if 35≤ BMI <40 and Obese III if 40≤ BMI.

Supplementary Table S2. Distribution of time since last cervical screening in relation to participant characteristics

Category	Less than 1 year	Between 1 to 2 years	More than 2 years	Never
**Self-rated health**				
Very Poor	6 (28.6%)	5 (23.8%)	10 (47.6%)	0 (0.0%)
Poor	85 (58.6%)	25 (17.2%)	29 (20.0%)	6 (4.1%)
Moderate	835 (54.1%)	315 (20.4%)	349 (22.6%)	45 (2.9%)
Good	1807 (62.6%)	549 (19.0%)	425 (14.7%)	107 (3.7%)
Excellent	340 (66.3%)	90 (17.5%)	61 (11.9%)	22 (4.3%)
**Age category**				
<25	108 (44.6%)	38 (15.7%)	20 (8.3%)	76 (31.4%)
25–34	582 (64.7%)	168 (18.7%)	103 (11.5%)	46 (5.1%)
35–44	924 (67.7%)	223 (16.3%)	196 (14.4%)	21 (1.5%)
45–54	987 (64.2%)	286 (18.6%)	250 (16.3%)	14 (0.9%)
55–64	374 (51.2%)	182 (24.9%)	165 (22.6%)	9 (1.2%)
65+	110 (29.5%)	95 (25.5%)	153 (41.0%)	15 (4.0%)
**Communication about cervical screening in your environment**				
Yes	2446 (63.9%)	718 (18.8%)	563 (14.7%)	98 (2.6%)
No	640 (48.4%)	274 (20.7%)	326 (24.7%)	82 (6.2%)
**Settlement type**				
Unknown	8 (88.9%)	1 (11.1%)	0 (0.0%)	0 (0.0%)
Capital city	699 (65.4%)	189 (17.7%)	148 (13.8%)	33 (3.1%)
Country-level city	904 (59.6%)	303 (20.0%)	259 (17.1%)	51 (3.4%)
Town	949 (59.8%)	303 (19.1%)	281 (17.7%)	54 (3.4%)

Large Village	96 (60.0%)	30 (18.8%)	28 (17.5%)	6 (3.8%)
Village	392 (53.8%)	155 (21.3%)	150 (20.6%)	31 (4.3%)
Abroad	12 (75.0%)	1 (6.2%)	0 (0.0%)	3 (18.8%)
**BMI category**				
Underweight	95 (59.0%)	32 (19.9%)	17 (10.6%)	17 (10.6%)
Normal	1410 (62.5%)	439 (19.5%)	316 (14.0%)	90 (4.0%)
Overweight	908 (58.7%)	296 (19.1%)	303 (19.6%)	41 (2.6%)
Obese I	458 (57.5%)	173 (21.7%)	151 (19.0%)	14 (1.8%)
Obese II	145 (56.4%)	42 (16.3%)	59 (23.0%)	11 (4.3%)
Obese III	58 (56.9%)	7 (6.9%)	31 (30.4%)	6 (5.9%)
**Physical activity**				
Not regularly	992 (55.9%)	355 (20.0%)	371 (20.9%)	56 (3.2%)
1–2 times a month	505 (59.1%)	158 (18.5%)	157 (18.4%)	35 (4.1%)
Once a week	583 (64.6%)	171 (19.0%)	110 (12.2%)	38 (4.2%)
Several times a week	840 (65.5%)	241 (18.8%)	168 (13.1%)	33 (2.6%)
Daily	168 (50.0%)	67 (19.9%)	83 (24.7%)	18 (5.4%)
**Smoking status**				
Non-smoker	809 (19.0%)	691 (16.2%)	146 (3.4%)	2615 (61.4%)
Smoker	184 (20.7%)	200 (22.5%)	35 (3.9%)	470 (52.9%)

Data is shown as absolute numbers and percentages in brackets, as n(%). Obese I if 30< BMI <35, Obese II if 35≤ BMI <40 and Obese III if 40≤ BMI.
